# Riverine Women’s Perceptions of the Pap Smear Test in Light of Health Literacy

**DOI:** 10.3390/healthcare14020175

**Published:** 2026-01-09

**Authors:** Thaynara Cordeiro Mendes, Letícia Calandrine Chagas, Marcio Yrochy Saldanha dos Santos, Ingrid Bentes Lima, Breno Augusto Silva Duarte, Ivaneide Leal Ataíde Rodrigues, Evelin Lorena Sousa do Espírito Santo, Paula Gisely Costa Silva, Laura Maria Vidal Nogueira

**Affiliations:** 1 Graduate Nursing Program, Universidade do Estado do Pará, Belém 66095-015, Brazil; thay.mendes02@gmail.com (T.C.M.); calandrini25@gmail.com (L.C.C.); 2 Undergraduate Nursing Program, Universidade do Estado do Pará, Belém 66095-015, Brazil; ingridbentes@outlook.com (I.B.L.); duartebreno30@gmail.com (B.A.S.D.); ivaleal2016@gmail.com (I.L.A.R.); evelin_santo@hotmail.com (E.L.S.d.E.S.); paula_gisely@outlook.com (P.G.C.S.); lauramavidal@gmail.com (L.M.V.N.)

**Keywords:** rural health, rural population, disease prevention, uterine cervical neoplasms, health literacy

## Abstract

**Background:** Women living in riverine communities are affected by factors such as geographical and cultural distance that hinder access to and use of health services. In this context, access to the Pap smear is crucial for the early detection of cellular changes that may progress to cervical cancer, which underlines the importance of understanding riverine women’s subjective perceptions of this exam. **Objectives:** To analyze the perceptions of riverine women regarding cervical cancer screening through the lens of health literacy. **Methods:** Descriptive qualitative study conducted with 42 riverine women residents of the Brazilian Amazon who were registered at the Basic Health Unit on Cotijuba Island, Pará, Brazil. Data were collected through semi-structured individual interviews from January to May 2024 and analyzed using IRaMuTeQ software version 0.7 alpha 2. **Results:** Data were grouped into similar classes, yielding the following thematic axes: knowledge, feelings and perceptions about the Pap smear test; how health literacy and access to information affect self-care; access to health services. The study showed that limited participant knowledge about the Pap smear was reflected in low health literacy, which directly affected adherence to the exam. **Conclusions:** The study demonstrated that the riverine woman’s limited knowledge regarding the Pap smear was reflected in their poorly developed health literacy, which directly contributed to non-adherence to the exam.

## 1. Introduction

More than 500,000 cases of cervical cancer (CC) occur worldwide each year, with a higher burden in less developed countries [1]. In Brazil, the estimated number of new cases per year for the 2023–2025 triennium was 17,010, corresponding to a crude incidence rate of 15.38 cases per 100,000 women; cervical cancer is the third most incident cancer among women and was ranked fourth in mortality in the country in 2021 [2].

Regarding CC mortality, Brazil recorded 7209 deaths in 2023. In the Northern region, 944 deaths were reported, of which 402 occurred in the state of Pará, representing approximately 42.5% of female cancer deaths in the region [3]. In this context, actions aimed at early diagnosis should be prioritized as essential strategies to reduce the incidence and mortality of this disease [4].

Cervical cancer screening is performed through the Preventive Cervical Cancer Exam (PCCU), commonly known as the Pap smear, which is the principal method for early detection of cervical cellular abnormalities. It is a simple, painless test offered across the public health network for women aged 25 to 64 years who have initiated sexual activity [2].

According to the Cancer Information System (SISCAN), by July 2025, a total of 3,433,851 cytopathological tests had been performed in public health units across Brazil. Of this total, 336,104 were performed in the Northern region and 141,353 in the state of Pará alone [5]. Within the priority age group for screening (25–64 years) as established in national guidelines, data for the Northern region recorded 640,991 women screened by the Unified Health System (SUS) in 2022. This figure represents one of the lowest coverage rates in the country, highlighting weaknesses in the access and effectiveness of preventive actions in the region [2].

This scenario of low screening coverage, together with high incidence and mortality rates, underlines the need to implement strategic preventive actions, such as health education grounded in women’s Health Literacy (HL) levels [6]. Health literacy refers to the cognitive competencies that determine the motivation and ability to obtain, understand, and use information for self-management of health—in this study, to undergo the Pap smear regularly [7,8].

Opportunities to access reliable information vary across populations, leading to different HL levels. Determinants of HL include living conditions, access to education, and availability of health services. Among population groups with greater HL limitations, riverine communities stand out, facing high levels of social exclusion, poor living conditions, low educational attainment, and restricted access to health services [1].

Low levels of HL are associated with lower therapeutic adherence and difficulties in self-management of care [9]. Moreover, although patient autonomy is fundamental to the therapeutic process, self-management has proven to be ineffective for a significant portion of the population [10]. A direct relationship between HL and health behaviors is also observed, as the level of HL influences daily decisions related to self-care [11].

Women living in riverine communities experience intense social and economic inequalities compared with women in urban areas [12]. They face geographical barriers, economic disparities, and inadequate health services [1], which compromise their access to basic information about cervical cancer. In addition, local cultural norms may discourage seeking preventive care, such as the PCCU, as well as other essential services [13].

This study was guided by the following research question: What are the perceptions of riverine women regarding the Pap smear and the factors influencing adherence to the exam? The objective was to analyze the perceptions of riverine women about cervical cancer screening in light of health literacy, which allowed the identification of intrinsic factors related to the subjectivities expressed in the behavior of riverine women concerning test uptake.

## 2. Materials and Methods

### 2.1. Study Design

Descriptive qualitative study guided by the Consolidated Criteria for Reporting Qualitative Research (COREQ) framework [14]. The concept of HL, as proposed by Nutbeam [7], was adopted to explore the perceptions surrounding the Pap smear.

### 2.2. Study Setting and Recruitment

The study was carried out at a Basic Health Unit (UBS) on Cotijuba Island—an insular area of Belém, the capital of Pará, in the Brazilian Amazon—located 22 km from the city center, with an estimated population of around 10,000 inhabitants [15].

### 2.3. Participants, Inclusion and Exclusion Criteria

Forty-two riverine women participated in the study. Eligibility criteria included being aged 18 years or older, residing on the island, and being registered with the local BHU, regardless of whether they had previously had a Pap smear. No participants were excluded.

### 2.4. Data Collection

Data were produced between January and May 2024 through individual semi-structured interviews containing five open questions about the Pap smear and health literacy, aiming to explore women’s subjectivities on the topic and their possible information sources, as well as facilitators and barriers in their relationships with the health team. Participants were randomly selected upon presenting at the UBS. Upon providing written consent, they were identified by the letter ‘E’ (representing ‘Interview’), followed by a sequential Arabic numeral. Interviews were audio-recorded with digital devices, transcribed verbatim, and stored in Microsoft Word 2021. Interviews were concluded when saturation was reached and collecting additional information no longer changed the understanding of the phenomenon studied [16].

The interview was conducted using a guide comprising five open-ended questions that explored perceptions regarding the Pap smear, information sources, the importance attributed to the exam, and the location where the exam had previously been performed, as well as whether participants had encountered difficulties in understanding, accessing, or using information about the Pap smear.

### 2.5. Data Analysis

Data were analyzed using lexical analysis with the Interface de R pour les Analyses Multidimensionnelles de Textes et de Questionnaires (IRaMuTeQ), version 0.7 alpha 2, which enables several statistical text analyses, including Descending Hierarchical Classification (DHC) [17]. DHC was applied in this study and represented graphically as a dendrogram indicating word classes and their interrelations. From the classes generated by DHC, associations among statistically significant words (*p* < 0.05) and text segments of each class were interpreted to determine the meaning assigned to each class. The classes were organized into thematic axes for clearer comprehension of the contexts present, following the partitioning logic of the corpus.

### 2.6. Ethical Considerations

This study was approved by the Research Ethics Committee (CEP) of the Magalhães Barata School of Nursing at the State University of Pará under opinion No. 6.152.465. Participation was voluntary, and all participants provided written informed consent. The collected data preserved participant anonymity. The study followed the ethical guidelines set out in Resolution 466/2012 of the National Health Council of the Brazilian Ministry of Health for research with human beings, and it was also grounded in the Nuremberg Code and bioethical principles, ensuring voluntary consent, social benefit, scientific basis, and freedom to withdraw for all participants.

## 3. Results

### 3.1. Participant Characteristics

Regarding socioeconomic profile, most women were aged between 25 and 62 years (n = 31; 81.58%), lived in conjugal union (n = 28; 73.69%), had completed secondary education (n = 16; 42.11%), had children (n = 31; 81.58%), and had undergone a Pap smear, with the highest proportion reporting having undergone the test in 2023 (n = 16; 42.11%).

### 3.2. Characterization of the Textual Data

**Figure 1:**
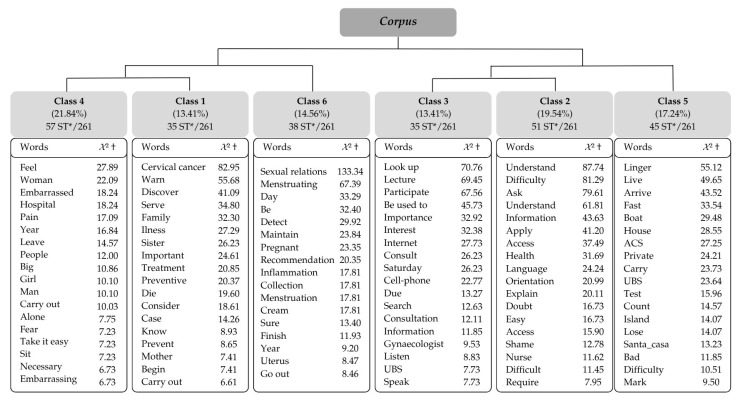
Dendrogram of the Descending Hierarchical Classification derived from the interviews using IRaMuTeQ software. * = Text segment, † = Chi-square.

The corpus consisted of 42 texts, divided into 329 Text Segments (TS), of which 261 (79.33%) were retained, resulting in six classes by DHC. The corpus was subdivided into two subcorpora: the first composed of classes 4, 1, and 6, and the second composed of classes 3, 2, and 5 (Figure 1).

It is noteworthy that the construction of the thematic axes was based on the classes in the following order: 4, 1, 6, 3, 2, and 5, from left to right. The partition logic generated by IRaMuTeQ processing divided the corpus into two subcorpora. Classes 4, 1, and 6 were aggregated into one subcorpus due to the words presenting complementary contexts among these classes. In the other subcorpus, classes 3, 2, and 5 were aggregated. The thematic axes and their respective classes are presented below.

#### 3.2.1. Thematic Axis 1—Knowledge, Feelings, and Perceptions About the Pap Smear Test (Classes 6, 1, and 4)

Class 6 highlights words such as “sexual relations”; “menstruating”; “day”; “detect”; “recommendation”; “inflammation”; “cream”, which reflect healthcare team recommendations for preparing for the exam.

“*[…] To have it done I know you must not have had sexual intercourse, not be menstruating and not be using those vaginal creams*”

(E10)

“*I know that before the Pap smear you should refrain from sexual intercourse for three days and not be menstruating*”

(E29)

Class 4 includes words such as “feel”; “embarrassed”; “pain”; “year”; “leave”; “man”, emphasizing feelings experienced during the PCCU and factors identified as determinants of non-adherence or irregular attendance.

Most participants reported feeling embarrassed and ashamed when undergoing the Pap smear due to exposing their genitals to health professionals, particularly when the professional was male. This is linked to a lack of trust in professionals and fear that intimate information could be revealed by others. When the examination was performed by a professional of the opposite sex, feelings of shame and fear regarding the procedure were exacerbated, often stemming from the husband’s lack of consent. These cultural and gender norms constitute factors that may compromise cervical cancer screening.

“*[…] Once I didn’t do it because it was a man; when it’s a woman we feel more at ease*”

(E16)

“*[…] One time I was waiting and I left to wash my hands and overheard something I shouldn’t have—the nurse talking to the woman who had the test, saying she was going to vomit because the woman hadn’t bathed; that’s unethical. I said so and I didn’t go in, I went home*”

(E27)

Fear of pain during the procedure and anxiety about a possible positive cancer diagnosis were other emotions reported, causing some women to postpone or avoid the PCCU. This attitude reveals a lack of knowledge about the importance of early detection.

“*[…] It’s been two years since I had it done; I’m scared and I don’t like it, you feel discomfort at the time*”

(E35)

“*[…] Many women are afraid of the result*”

(E5)

This class also revealed uncertainty about the recommended screening interval.

“*I know you have to have the Pap smear every three months*”

(E12)

“*I do it annually, but I have heard it is every six months*”

(E30)

Words present in Class 1 reflect participants’ understanding of the exam’s purpose and perceptions related to undergoing the PCCU; the most representative words included: “cervical cancer”; “prevent”; “discover”; “serve”. This class also identified primary references and motivations for having the test, expressed in words such as “family”; “illness”; “sister”; “important”; “preventive”; “die”; “consider”.

Few participants demonstrated knowledge of the exam’s purpose, even among those who had undergone it regularly, indicating that women may submit to the test without knowing its main objective. Others associated it with disease prevention but expressed doubts about its importance.

“*I don’t know what the Pap smear is or what it’s for. I did it because my mother always said we should go to the health unit to take care of our bodies*”

(E30)

“*To be honest I don’t know what this preventive exam is because I’ve never had it, but I know it’s to prevent several diseases*”

(E26)

When asked about knowledge of cervical cancer, participants often showed uncertainty regarding its primary causes, although some recognized its symptoms.

“*[…] How is cervical cancer caused, where does it come from? That’s my question*”

(E17)

“*Cervical cancer is a strong bacterium that spreads and, if not detected early, has no cure or treatment. A woman may have bleeding and pain*”

(E29)

Although most participants lacked knowledge about CC and the Pap smear, two women mentioned multiple sexual partners and human papillomavirus (HPV) as causal factors.

“*[…] Women who have an active sexual life, many partners, are more at risk for this disease; that’s why it’s very important to have this preventive test*”

(E02)

“*[…] Besides genetics, HPV, syphilis, small discharges are a major warning sign*”

(E27)

Even with limited knowledge about CC and the Pap smear, all participants considered the test important. Fear of death and the consequences of the disease, influenced by the experiences and accounts of friends and relatives—such as grandmothers and mothers affected by cancer, often with late diagnoses—were major motivations for adhering to screening.

“*I consider it very important to do the Pap smear because I had a friend who died of cervical cancer. She was diagnosed very late, so I prefer to come and have it done; I never skipped it*”

(E18)

“*I talked to my mother about the Pap smear; my aunt had cervical cancer and she never used to do the Pap smear; when she finally did she was already in pain and very ill, she started treatment*”

(E03)

Participants’ recognition of the importance of early diagnosis through the test was expressed in accounts indicating access to better therapeutic measures and improved prognosis.

“*Twenty years ago my mother had the Pap smear and they found cervical cancer; she completed all the treatment and today she is fine, so it’s important to do it; that’s why my sisters and I do it every year*”

(E37)

“*[…] I consider the Pap smear important because if my grandmother had done it maybe the cancer would have been discovered earlier and she wouldn’t have died*”

(E41)

#### 3.2.2. Thematic Axis 2—How Health Literacy and Access to Information Affect Self-Care (Classes 3, 2, and 5)

Class 3 reveals how health information is accessed. Words such as “internet”; “cellphone”; “search”; “consult”; “information”; “UBS” illustrate information sources, including health unit educational talks, online searches, and conversations with health professionals.

Among the means mentioned, educational activities at the health service stood out as an important venue for disseminating the importance of the test and motivating women to have it, acknowledging that a lack of knowledge keeps them from engaging in self-care.

“*I don’t have difficulty understanding information; I become aware when information is detailed, but it would be important to hold talks about the Pap smear and its importance for our health; this would help increase the number of women coming to do the test*”

(E10)

“*I’ve never had the Pap smear, so I don’t know what needs to be done beforehand. I never sought information about it*”

(E1)

Participants’ accounts also make it clear that certain everyday situations prompt them to seek the PCCU, such as the appearance of symptoms or disease diagnoses among their families or social circles. Nevertheless, difficulty accessing health services is a barrier to timely testing. Moreover, online consultation is hindered by a lack of internet access.

“*[…] The difficulty is getting to the health unit; like I said, it depends on the boat—if there is a boat you can come*”

(E02)

“*I used to have trouble accessing information because we didn’t have a cellphone or access to information; now that they installed Wi-Fi it’s better, but I still have trouble understanding and applying it*”

(E22)

Class 2 highlights participants’ health literacy and presents different levels of comprehension for health promotion and how this skill influences the use of health services and personal health care. Representative words for this class include: “understand”; “difficulty”; “ask”; “information”; “explain”; “access”; “language”; “doubt”; “access”.

When asked about difficulties obtaining, understanding, and using health information, participants generally reported easy comprehension, but noted challenges when technical terms were used. They emphasized that when information is communicated clearly and opportunities are provided to clarify doubts, understanding is easier.

“*I don’t have difficulty accessing, understanding and applying information because when they explain properly we understand. When I have time I research more deeply. Some health professionals use accessible language; when they speak in a difficult way I complain because otherwise I leave without knowing anything*”

(E27)

“*I don’t have difficulty understanding the Pap smear information; when I realize I don’t understand, I ask so I don’t do it wrong*”

(E07)

If the health team does not attend to women’s information needs, educational actions will not positively influence adherence to CC screening. Difficulty applying recommendations can also stem from feelings of embarrassment; when women do not understand an explanation but are ashamed to ask, this hinders self-care.

“*I think it would be important to explain why the Pap smear is necessary and what it means. They just give a paper and don’t explain anything*”

(E14)

“*I have all the difficulty accessing information because I’m ashamed to ask about it. I can’t understand either; I think health professionals’ language is a bit difficult and I’m ashamed to ask, so I can’t do anything*”

(E32)

#### 3.2.3. Thematic Axis 3—Access to Health Services (Class 5)

Class 5 expresses participants’ difficulties accessing the health unit and factors that lead them to seek the test outside the island’s unit. Testimonies predominantly include the words: “linger”; “arrive”; “live”; “fast”; “private”; “test”; “boat”; “Community Health Worker (ACS)”; “UBS”; “island”.

Access difficulties primarily affect women living on smaller neighboring islands around Cotijuba Island due to a lack of public transport between islands, imposing costs they cannot always afford. The tide movements (high and low tides) can also limit boat traffic.

“*Many women from where I live don’t come because it’s very difficult to get to the health unit. You have to take a boat, you need a small boat to come; sometimes the weather is bad, lots of spray and storms. To get here today, we left the island at eight and arrived at nine thirty*”

(E38)

“*When I had mine done I did it at the hospital, but I haven’t done it here at the Cotijuba health unit because it’s difficult for me to get here; I live far away so transportation is very difficult; you have to pay ten reais to go and come back and sometimes we don’t have it, so all that makes the situation difficult*”

(E12)

Women living on Cotijuba Island itself also reported difficulties getting to the unit, primarily due to its distance from their homes.

“*Because I live far away, it takes me a long time to get to the health unit; I arrive in about an hour to an hour and a half because of the road*”

(E22)

“*[…] I live far from the health unit; it takes me half an hour to an hour to get here*”

(E26)

Some participants reported having the PCCU done in the private network due to delays in receiving results at the island health unit.

“*I usually have the Pap smear quite frequently; the last time I had it was in November 2023. I don’t do it at the Cotijuba health unit because sometimes the result takes too long, so I prefer to see a private physician—it’s faster*”

(E11)

“*I always had the Pap smear privately because I wanted everything faster and because I work and don’t have time to be here, so I preferred private care*”

(E29)

## 4. Discussion

The findings indicate limited knowledge among riverine women about the Pap smear, reflecting insufficient health literacy. Inadequate understanding leads to superficial and stigmatized views of the test and cervical cancer, compromising adherence and regular uptake of the Pap smear. In addition, difficulties accessing services and health information contribute significantly to low screening coverage, especially in remote areas.

Although some participants were familiar with the PCCU, they did not understand its true purpose. A similar observation was reported in a study conducted in Wolaita Zone, Ethiopia [18], where most participants had never heard of cervical cancer and did not know the preventive purpose of the Pap smear. Although the study was conducted in a different country, similarities with the riverine population of Cotijuba are evident, as this group faces limitations in their access to health services, significant educational challenges, and high population growth.

Education level has been identified as a factor influencing women’s adherence to screening; women with higher education tend to undergo the test more readily as they value seeking information and using health services for prevention. Conversely, women in socioeconomically and educationally disadvantaged situations face obstacles accessing services and understanding information about the test, limiting self-care due to a lack of knowledge about the disease and the importance of screening [19].

Access to relevant information can facilitate a greater understanding and application of preventive practices, thereby enhancing self-care, including participation in PCCU. The task for health services must expand beyond problem-solving to include prevention and health promotion [20]. Therefore, informing women about cervical cancer, its risk factors, and preventive measures is essential.

Emotional barriers such as the fear of the result, pain during the procedure, shame, and embarrassment hinder adherence and correct periodicity of the test among riverine women; these feelings are stronger when the exam is performed by male professionals. A study in India found similar barriers to cervical cancer screening, indicating that these feelings are common among women across different regions of the world [21].

Regarding the maintenance of testing regularity, there was uncertainty even among those who had undergone the exam, leading to intervals different from those recommended by the Ministry of Health—a reflection of insufficient guidance from health professionals that may result in unnecessary repeat testing [19].

A positive finding of this study was that almost all women could identify and report following two or more preparatory care measures before the exam, and understood the purpose of these measures. This contrasts with a study in Maranhão where 86.2% of respondents could not identify any preparatory recommendations. Observing proper preparation is fundamental to obtaining a higher-quality sample and, thus, more accurate results [20].

The study indicates that preventive behavior is often triggered only by the appearance of symptoms or by the experience of disease within family or social circles rather than by routine preventive practice. This underscores the need for health education to encourage the use of services, not only for treating gynecological problems, but primarily for prevention and health promotion [22].

With regard to educational activities to disseminate information about the Pap smear, the health team on the island was recognized for its efforts to offer health education to construct knowledge. Education contributes to autonomy in individual and collective care, transforming behaviors and potentially increasing adherence to cervical cancer screening. Health education is therefore a relevant pedagogical process that promotes critical and reflective perspectives on women’s health realities and clarifies the importance of screening to foster autonomy in health decision-making and self-care [7].

Noteworthy is the use of digital consultation as an information-seeking strategy among riverine women, since the internet provides rapid access to various content and may contribute to a better understanding of health issues. However, unequal internet access presents a challenge for digital inclusion, as a significant portion of the population lacks the necessary resources to access new technologies. Consequently, digitally excluded individuals have reduced opportunities to obtain information and transform it into knowledge [23].

Access to information among riverine women facilitated knowledge acquisition, but HL limitations remained. Factors such as poor communication with health staff and difficulty understanding complex concepts interfere with assimilating professional guidance. An educational intervention for cervical cancer screening among rural Chinese women emphasized that inadequate HL significantly affects disease prevention and highlighted the importance of providing information adapted to women’s needs [24].

In the riverine female population, HL is a major challenge due to community-specific characteristics, including adversities in accessing information that enable informed decision-making. A quantitative study conducted in Portel (Mesorregião do Marajó, Pará) found results similar to the present research, attributing low healthcare seeking to educational, socioeconomic, and geographic factors. Heterogeneous and culturally adapted approaches are needed to mitigate challenges and promote effective, accessible health for these populations [25].

In addition to the difficulties already described, access to health services for preventive exams is hampered by geographic distance from homes to health units, compounded by the absence of public transportation and lack of financial resources to pay for private transport. This finding mirrors a Malawian study that identified substantial social inequalities in rural areas compromising access to PCCU because of geographic barriers and poor or absent transport [26].

Consequently, empowering riverine women for health self-management involves both an individual willingness to seek information and care and the quality of relationships with health services and professionals. Health professionals, especially nurses, should develop efficient educational actions about the PCCU to ensure a clear understanding of all aspects of the test, contributing to regular adherence, correct testing intervals, and identification of barriers that prevent regular, responsible prevention practices, including emotional factors experienced during sample collection [27].

A study conducted in Turkey identified that low levels of HL influence women’s self-care [28]. This aligns with the results of the present study, demonstrating that women’s limited access to health information leads to lower levels of HL and, consequently, impacts their self-care, as it reflects their capacity to understand information and apply it in their daily lives.

Based on these findings, health interventions utilizing health promotion strategies aimed at stimulating the skills inherent to the HL of riverine women will be capable of strengthening self-care, mitigating potentially harmful perceptions regarding the exam, and enhancing these women’s access to healthcare. Furthermore, the health professional plays a fundamental role in providing access to health information, facilitating understanding, and encouraging the use of this information, thereby contributing to informed health decision-making, empowerment, and self-care among riverine women.

## 5. Conclusions

It is concluded that riverine women’s perceptions of the Pap smear stem from insufficient health literacy. Limited HL directly impacts self-care and women’s ability to make informed, safe, and effective health decisions. Although health activities are carried out sporadically, there is a clear need to develop more effective preventive and educational measures to improve the uptake and regularity of cervical cancer screening, taking into account social determinants and the main barriers that impede access to and use of preventive services.

Therefore, further studies on this topic are essential, as qualitative research on HL remains scarce in Brazil. With a larger body of evidence, it will be possible to profile subjective factors that hinder increased screening coverage and, consequently, enable reductions in cervical cancer incidence rates in Brazil. Moreover, the Amazonian context of this research provides unique evidence for tackling the problem in territories with particular geographic and cultural specificities.

## Data Availability

The original data presented in this study are included in the article. Further inquiries can be directed to the authors.

## Abbreviations

The following abbreviations are used in this manuscript:

ACS	Community Health Worker
CC	Cervical Cancer
CEP	Research Ethics Committee
COREQ	Consolidated Criteria for Reporting Qualitative Research
DHC	Descending Hierarchical Classification
HL	Health Literacy
HPV	Human Papillomavirus
IRaMuTeQ	Interface de R pour les Analyses Multidimensionnelles de Textes et de Questionnaires
PCCU	Cervical Cancer Preventive
SISCAN	Cancer Information System
SUS	Unified Health System
UBS	Basic Health Unit
